# Vallecular cysts in clinical practice: report of two cases

**Published:** 2016

**Authors:** A Zamfir-Chiru-Anton, DC Gheorghe

**Affiliations:** * Department of Otorhinolaryngology, “G. Alexandrescu” Children Emergency Hospital, Bucharest, Romania,; ** Department of Otorhinolaryngology, “M.S. Curie” Children Clinical Emergency Hospital; “Carol Davila” University of Medicine and Pharmacy, Bucharest, Romania

**Keywords:** vallecular cyst, children, surgery, treatment

## Abstract

The objective of the two case reports was to discuss the pathogenesis, origin, and therapeutic options for vallecular cysts.

**Method of Study.** 2 children diagnosed in our department were treated by transoral endoscopic surgery, using cold instruments, suspension laryngoscopy and the operating microscope.

**Results.** Both cases presented breathing and deglutition problems at diagnosis. The surgery aimed the complete excision, with cold instruments, via transoral access. No complications occurred and the postoperative healing was uneventful. No recurrences were observed on the long-term follow-up.

**Conclusion.** Clinical, imagistic, and surgical data support the thyroglossal duct origin of vallecular cysts (base of tongue variant) and the complete excision is required to obtain long lasting cure.

## Introduction

Vallecular cysts have been described in the literature for some time [**1**]. From an anatomical point of view, there are laryngeal cysts (that develop in close contact with the anterior face of the epiglottis, named true vallecular cysts by some authors) and base of tongue cysts (sometimes reported as vallecular pseudocysts). These lesions could have a different pathogeny and, hence, different therapeutical approaches [**2**]. Due to the paucity of cases presented in the literature, the incidence of the disease is unknown. It can determine respiratory obstruction and stridor or dietary intake difficulties with failure to thrive in neonates [**3**]. Their origin is debatable, most of the theories being malformative [**4**]. The reason for discussing the pathogenesis of such cysts is linked to the best therapeutical option and the investigation taken into consideration when approaching these cases. Their diagnosis is relatively easy, using nasopharyngeal laryngoscopy (NPL). Treatment techniques include: marsupialization [**5**] and surgical excision (with/ without CO2 laser) [**6**]. Some authors described the use of coblation for the excision of the cyst [**7**].

## Case report

**Fig. 1:**
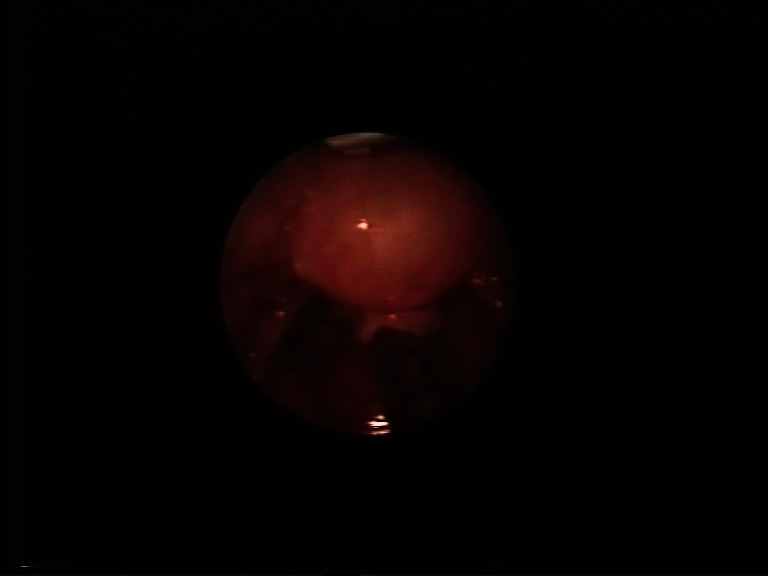
Preoperative view of case 1

**Fig. 2:**
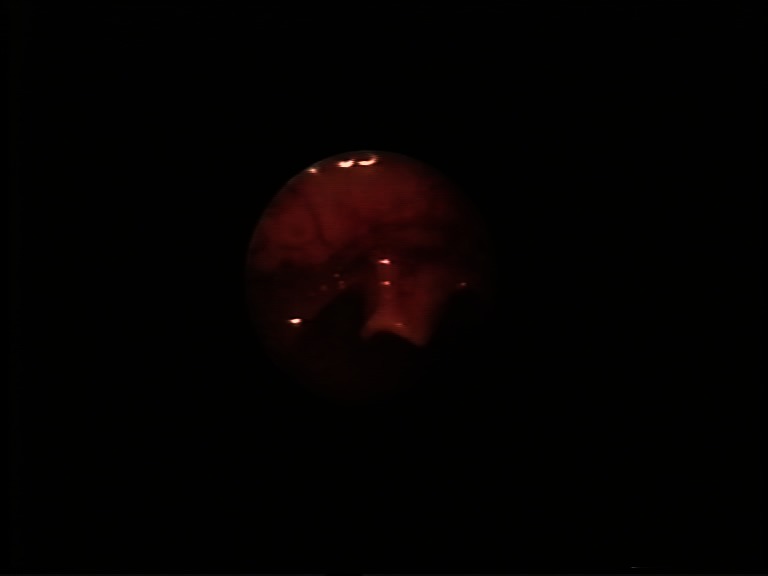
Postoperative view of case 1

A ten-year-old boy presented in our department with deglutition problems, intermittent odynophagia, and subtle dysphony. NPL and physical exam showed a median lump at the base of tongue, covered by normally colored mucosa (**Fig. 1**). Ultrasonography (US) verified the presence of the normal thyroid gland in the cervical region. CT-scan was performed to verify the dimensions of the cyst and its relations with the surrounding structures. Cold instrument excision was performed under general anesthesia (GA) and suspension laryngoscopy, followed by the local cautery of the wound. Healing occurred uneventfully, with analgesics and antibiotics administered postoperatively. The histologic exam revealed a cystic tumor lined by stratified cuboidal epithelium and moderate lymphocytic infiltration. The 2-year follow-up showed no sign of local recurrence (**Fig. 2**).

**Fig. 3:**
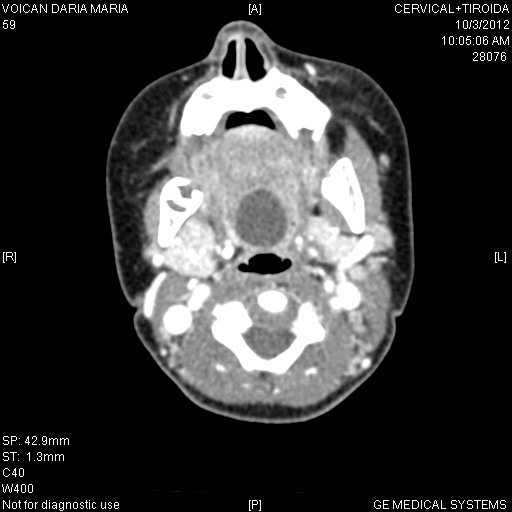
Preoperative CT imaging of case 2

**Fig. 4:**
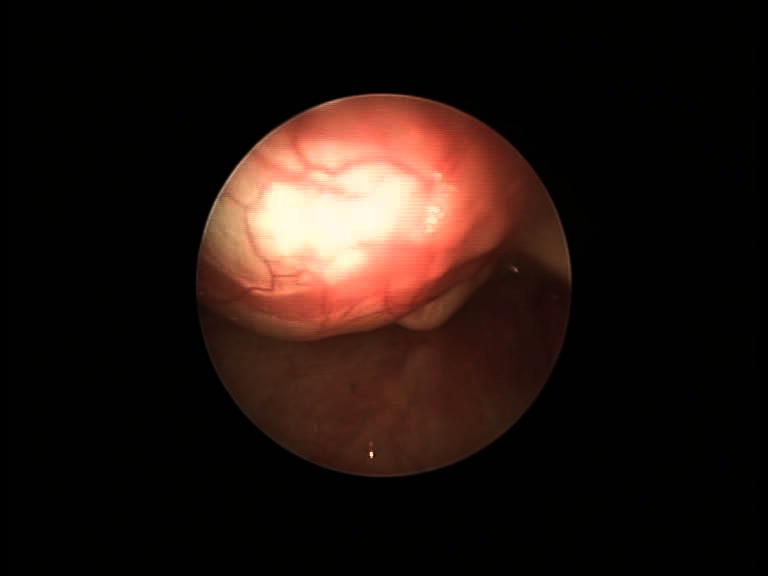
Preoperative view of case 2

**Fig. 5:**
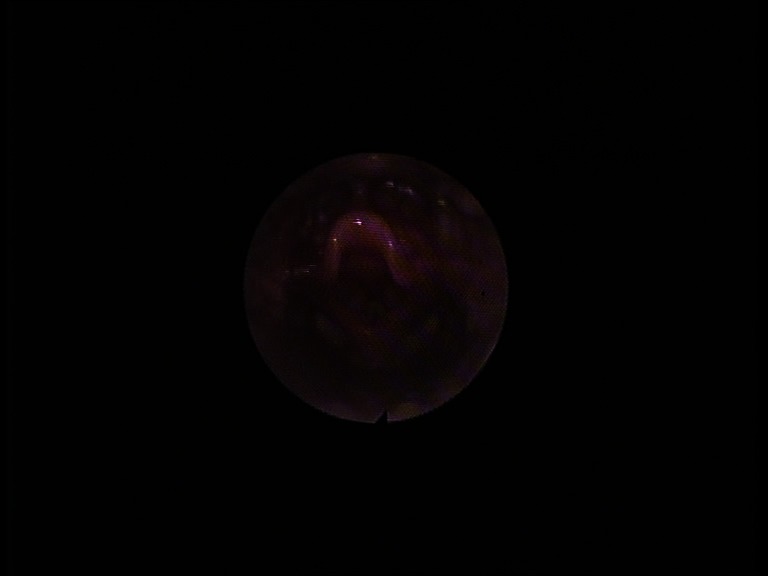
Postoperative view of case 2

A second case involved a girl aged 1 year and 8 months, who presented with frequent food aspiration into her lower airways, needing prolonged and recurrent hospital admission. Minor respiratory stridor was sometimes present when supine. Neurological problems were suspected but no other handicap was obvious. NPL and physical exam revealed a median tumor at the base of the tongue (**Fig. 4**). Cervical US showed a normal thyroid gland. CT-scan was performed in order to demonstrate the cyst dimensions and its position (**Fig. 3**). The cyst was completely excised under GA and by using suspension laryngoscopy. No suture of the remaining wound was applied. Histological sections showed the presence of a cystic cavity lined with cuboidal stratified epithelium and minor inflammatory infiltrate. Healing recorded no complications. The patient was seen at 1-year follow-up with no recurrence of the cyst (**Fig. 5**). 

## Discussion

Vallecular cysts have been described under different names in the literature: epiglottic cysts, ductal cysts, etc. They consist of a liquid-filled median lump located at the base of the tongue, close to the epiglottis. They are covered with normal appearing mucosa. On the other hand, laryngeal cysts have their origin on the mucosa of the anterior face of the epiglottis and develop towards the vallecula [**2**]. Our opinion is that it is important to make the differential diagnosis between these lesions. Both cysts can produce stridor, dyspnoea, deglutition troubles and failure to thrive in neonates [**8**]. Symptoms can be found at any age but can be critical in neonates [**9**]. The diagnosis is relatively simple, with NPL, cervical region ultrasonography and CT-scan. Sometimes the Tc 99m cervical scintigram can be useful [**7**]. 

Our patients presented with base of the tongue variety cysts (vallecular pseudocysts). There was no unique hypothesis upon their origin. Some considered the possibility of a large mucous gland located into the thickness of the lingual tonsil. We considered this a less likely origin because the region was mainly lymphoid in its histology, with few secretory glands. Moreover, the existence of constant local, mechanical trauma/ pressure (from food/ deglutition) did not favor the blockage of local mucinous glands but their drainage. That is why we considered this kind of cyst a form of thyroglossal duct remnant. That would also explain the perfect median location, as seen on the CT-scan images and the observed tendency to develop towards the anterior muscular region of the tongue and the hyoid bone. 

The normal appearance of the cyst covering mucosa and the close adherence of the cyst lining to the muscular fibers of the tongue, with no preexisting dissection plane, were also noticed during surgery. 

The CT-scan also showed the profound muscular and the strict median position of the cyst. All the data from imagistics and surgery suggested the thyroglossal duct/ cyst theory as a valid one, even if no proofs of active thyroid tissue could be histologically found inside the cysts. 

Treatment options in the literature consider marsupialization but mention the risk of recurrence. That is also quite possible if a thyroglossal duct cyst is considered and treated. In addition, that is why an excision was decided to be performed in both cases, although endoscopically. Along with suspension laryngoscopy, the operating microscope can offer the tools to safely remove all cyst lining and, as such, prevent recurrences.

## Conclusions

Vallecular cysts are different from laryngeal cysts. The term pseudocyst is more likely to reflect this condition and explains the lack of a real epithelial lining of the cyst. Their most likely origin is thyroglossal duct remnants. Consequently, their treatment should be guided by the same principles, complete surgical removal. The lack of a complete excision can explain recurrences. We believe that surgical excision should be the standard method, with an endoscopic approach, which yields the best results. We do not believe that the nature of the used instruments (cold instruments vs. laser) influences the outcome and rate of recurrence. Preoperative CT-scan is useful in verifying the adherence of the cyst to the hyoid bone. Those cases would better benefit from a cervical approach.

**Funding**

None. 

**Disclosure**

None. 

**Conflict of interest**

The authors declare that there is no conflict of interest regarding the publication of this paper. 

**Acknowledgement**

None. 
